# Length at Maturity, Sex Ratio, and Proportions of Maturity of the Giant Electric Ray, *Narcine entemedor*, in Its Septentrional Distribution

**DOI:** 10.3390/ani12010120

**Published:** 2022-01-05

**Authors:** Xchel Aurora Pérez-Palafox, Enrique Morales-Bojórquez, Hugo Aguirre-Villaseñor, Víctor Hugo Cruz-Escalona

**Affiliations:** 1 Centro Interdisciplinario de Ciencias Marinas, Instituto Politécnico Nacional, Av. Instituto Politécnico Nacional S/N, Col. Playa Palo de Sta. Rita, La Paz 23096, Baja California Sur, Mexico; xapp39@gmail.com; 2 Centro de Investigaciones Biológicas del Noroeste, Av. Instituto Politécnico Nacional 195, Col. Playa Palo de Santa Rita, La Paz 23096, Baja California Sur, Mexico; emorales@cibnor.mx; 3 Instituto Nacional de Pesca y Acuacultura. Centro Regional de Investigación Acuícola y Pesquera, Calzada Sábalo-Cerritos S/N, Mazatlán 82112, Sinaloa, Mexico; aguirre_hugo@hotmail.com

**Keywords:** maturity, reproductive peak, sigmoid model, length structure

## Abstract

**Simple Summary:**

The size at which 50 percent of a fish population reaches sexual maturity is an important parameter of life history and is useful for setting conservation goals and fishing efforts. Based on 305 individuals in a population of giant electric rays, *Narcine entemedor*, collected in artisanal fisheries in the Bahía de La Paz, Mexico in its northern distribution over a 2-year period, females were larger than males, but males dominated the sex ratio. Total length at maturity for females was 55.87 cm with mature females present all year; there was no apparent seasonality in the reproductive pattern. Using these data sets, there appeared to be continuous annual reproductive activity.

**Abstract:**

The size at which a certain fraction of a fish population reaches sexual maturity is an important parameter of life history. The estimation of this parameter based on logistic or sigmoid models could provide different ogives and values of length at maturity, which must be analyzed and considered as a basic feature of biological reproduction for the species. A total of 305 individuals of *Narcine entemedor* (*N. entemedor*) were obtained from artisanal fisheries in the Bahía de La Paz, Mexico. For the organisms sampled, sexes were determined and total length (TL) in cm was measured from October 2013 to December 2015. The results indicated that the females were larger, ranging from 48.5 cm to 84 cm TL, while males varied from 41.5 cm to 58.5 cm TL. The sex ratio was dominated by males ranging from 45–55 cm TL, while females were more abundant from 60 to 85 cm TL. Mature females were present all year long, exhibiting a continuous annual reproductive cycle. The length at maturity data were described by the Gompertz model with value of 55.87 cm TL. The comparison between models, and the model selection between them, showed that the Gompertz model had maximum likelihood and smaller Akaike information criterion, indicating that this model was a better fit to the maturity proportion data of *N. entemedor*.

## 1. Introduction

The size at which a certain fraction of the fish population reaches sexual maturity is an important parameter of life history [1]. This information is relevant for demographic analysis, stock assessment, and providing information for fishery control rules, such as establishment of minimum legal length and closed fishing seasons [2,3]. In such analyses, one can achieve biological reference points, defined as metrics of stock statuses, such as fishing mortality values and biomass level [4,5].

The data used to estimate an appropriate size at sexual maturity should provide information on two aspects: (1) an observed proportion of physiologically mature individuals, meaning organisms capable of producing viable gametes, and (2) the proportion of these that are actually producing eggs at a given time [6]. Thus, the logistic model is most commonly used to describe the relation between body size and sexual maturity [1,7]. The plot of this model represents a proportion of mature females in each size class, consequently an S-shaped relationship with an asymptote approaching 1.0 for the largest sizes is commonly estimated. However, in some iteroparous species, not all females are physiologically mature during the reproductive season. In several cases reproduction occurs through batch fecundity (e.g., anchovies, sardines) and, for these species, the values of length at maturity are highly variable and the asymptote denoting the proportion of maturity will differ from 1.0 [6].

There are many methods for estimating the length at maturity, often referred to as the length at which 50% of the organisms are mature $\left(L_{50}\right)$. These include the models of Gompertz [8], Lysack [9], and more recently White et al. [10]. In many studies, the criteria for selecting a model are often arbitrary [11,12]. Therefore, the estimation of $L_{50}$ parameters and their precision in these models are based solely on a single average model [13]. To have a better and more robust approach, the model selection based on information theory and maximum likelihood theory is a relatively new paradigm in which several models are compared to each other, evaluating the support of the observed data with respect to each model [13,14]. Studies in reproductive biology using multimodel inference to estimate $L_{50}$ are scarcely reported in the literature, and this is mainly in teleost fishes, such as bigeye tuna (*Thunnus obesus*) [15] and herring (*Opistonema libertate*) [3]. In elasmobranchs, particularly from Mexican waters, only the shark, *Rhizoprionodon terraenovae* [12], and the bat ray (*Myliobatis californica*) [16] have been analyzed using the approach previously described.

According to the data observed, the estimations of $L_{50}$ based on logistic or sigmoid models could provide different ogives, such that the choice of the candidate model influences the expected $L_{50}$. In addition, its values could be biased whether the model used does not fitthe data set adequately, affecting the biological interpretation of the length at which 50% of the organisms are mature. This situation is crucial when the species exhibits viviparity, such as in several species of elasmobranchs. Biologically, the knowledge of $L_{50}$ does not indicate a maternity condition for all those females reaching this length. According to Walker [17] (pp. 81–127), the ogives of $L_{50}$ and the maturity condition are independent; particularly the $L_{50}$ could provide the beginning of the maternity condition. Therefore, the accuracy of $L_{50}$ is relevant because it would be indicating the females that are recruited to the reproductive stock. However, the adequate quantification of the number of births is necessary for the estimation of the size at maternity, which must be larger than the size at maturity. Hence, the first step is the estimation of an $L_{50}$ value that is sufficiently informative for simultaneously understanding the length at which 50% of the females are mature, as well as the beginning of their potential size at maternity. Consequently, the multimodel inference approach for estimating $L_{50}$ is a statistical procedure useful for this purpose.

According to this biological background, the giant electric ray, *Narcine entemedor* (Jordan and Starks, 1895), is identified as a viviparous species with a continuous annual reproductive cycle and limited histotrophy as a reproductive mode, exhibiting embryonic diapause [18]. The population is distributed from Bahía Magdalena, on the west coast of Baja California Sur to Peru, including the Gulf of California and Galapagos Islands [19,20]. This species is incidentally captured by artisanal fisheries in the Eastern Tropical Pacific and is bycatch from fisheries that target higher-valued teleost or crustaceans [21,22,23]. Given this incidental feature, the species has been poorly studied and there is limited biological information in the region. The information is mainly associated with descriptions about its reproductive biology, age, and growth, along with its food and feeding patterns; therefore, key population features based on quantitative analysis are necessary to understand the demography of this species. Thus, in this study, we reanalyzed the length at maturity for *Narcine entemedor* using a multimodel inference approach based on candidate models with different shapes, number of parameters, and biological assumptions.

## 2. Materials and Methods

### 2.1. Collection of Samples

A total of 305 individuals were obtained from artisanal fisheries in the Bahía de La Paz, which is located in the Gulf of California, Mexico between 24°07′ and 24°21′ latitude north and 110°17′ and 110°40′ longitude west. The individuals collected are very common and abundant in the Gulf of California; the species is not protected throughout its range, and is a very well-known commercial species. Additionally, all applicable international, national, and/or institutional guidelines for the care and use of animals were followed. In this study, experimental use of organisms was not required. Sex was determined and total length was measured (TL, cm) for all individuals sampled from October 2013 through December 2015. The maturity data of *N. entemedor* were taken from Burgos-Vázquez et al. [18].

### 2.2. Criteria for Evaluating Maturity

Maturity in *N. entemedor* individuals was defined as immature (0) or mature (1), with macroscopic characteristics using the criteria proposed by Burgos-Vázquez et al. [18]. For females, the total length and degree of vitellogenesis of the ovarian follicles in the ovary, as well as the anterior oviduct and uterus condition, were considered. Females that presented ovaries with translucent ovarian follicles ≤5 cm and abundant ovarian stroma, slight differentiation between the anterior oviducts and the uterus, and uteri between 0.2 cm and 1.2 cm wide without eggs or embryos were considered as immature. Females that presented ovaries with yellow ovarian follicles ≥6 cm, a uterus that was well differentiated from the anterior oviducts, with widths ≥1.3 cm, with or without eggs or embryos were considered as mature. Based on this microscopic evidence, the macroscopic criteria for defining the binomial classification (0,1) were validated such that the macroscopic and microscopic condition of the ovaries and uterus showed matches [18]. Consequently, the uncertainty associated to the binomial classification describing the observed length at maturity from the macroscopic characteristics of *N. entemedor* is negligible.

### 2.3. Sex Ratio

Sex ratio was calculated monthly. The sex ratios were compared using a chi-squared (X^2^) test, assuming that the sex ratio was 0.5. The null hypothesis was rejected if the X^2^ estimated value was greater than 3.84 (α < 0.05, df = 1) [24,25]. Additionally, the sex ratio was also represented for each 5 cm (TL) length class.

### 2.4. Length at Maturity

Length at maturity of females was estimated using a binomial code (immature = 0 and mature = 1), the data were modeled into two length-at-maturity models (Table 1). *Pi* was the estimated proportion of mature fish in size class *i, exp* refers to the exponent which is the number of times a number is multiplied by itself, *TL_i_* was the total length of size class *i*, *γ* was the rate parameter related to the speed of size change from non-reproductive to reproductive status,$\;L_{50}$ was the length at which 50% of the organisms were mature, *ε* was the maximum proportion of maturity reached, $L_{95}$ was the length at which 95% of the organisms are mature, and µ was the amplitude of the maturity ogive. The WHI equation was modified, expressing it as a three-parameter function for modeling changes in the proportion maturity; thus, the *ε* parameter varied as follows: 0 ≤ *ε* ≤ 1, which allowed for the maximum fraction of mature females to be less than 1 [6].

The objective function for estimating the parameters in the candidate length-at-maturity models were fitted by minimizing the negative log-likelihood (−lnℒ) [26]:

$$-\ln ℒ=-\sum_{i=1}^{n}[m_{i}\times \ln(\frac{P_{i}}{1-P_{i}})+P_{i}\times \ln(k)]$$

where $n_{i}$ was the number of individuals in size class *i*, $m_{i}$ was the number of mature fish in size class *i*, and the quantity $\kappa =\left(\begin{array}{c} n_{i}\\ m_{i} \end{array}\right)$ was defined as the binomial coefficient and was computed as $\kappa =\frac{n_{i}!}{m_{i}!\times \left(n_{i}-m_{i}\right)!}$ Given that these models exhibited a correlation between parameters, estimates of confidence intervals (CI) in each model were obtained using the likelihood contour method [27]. A chi-squared distribution with df = 2 was used, such that values that were equal to or less than 5.99 were accepted within the CI [24]. The chi-squared estimator was [28]:

$$CI=2\left[-\ln ℒ\left(\theta_{est}\right)-\left(-\ln ℒ\left(\theta_{i}\right)\right)\right]\leq \chi_{df,1-\alpha }^{2}$$

where $-\ln ℒ\left(\theta_{est}\right)$ was the negative log-likelihood of the most likely value of $\theta_{i}$, $-\ln ℒ(\theta_{i})$ was the negative log-likelihood based on hypotheses of the value of $\theta_{i}$, $\chi_{1-\alpha }^{2}$ was the value of the chi-squared distribution with a confidence level of 1-α = 0.05 and *df* = 2 [28]. Model performance was evaluated using Akaike’s information criterion (AIC), where the best model was the one with the lowest AIC value [29,30].

## 3. Results

**Figure 1:**
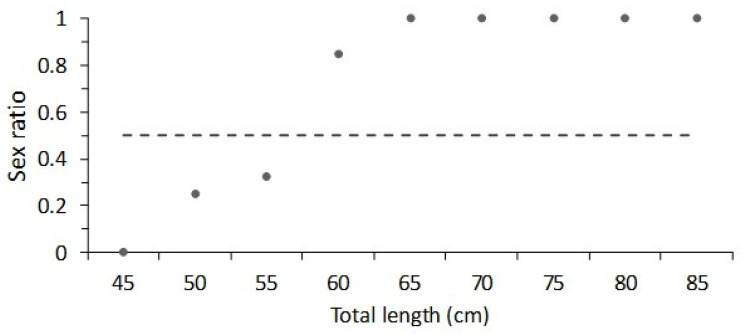
Sex ratio female:male (F:M) of giant electric rays, *Narcine entemedor*, by size intervals.

**Figure 2:**
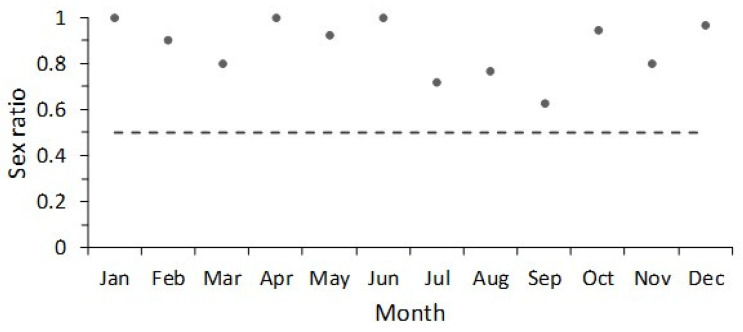
Monthly sex ratio (F:M) of giant electric rays, *Narcine entemedor*.

In total, 260 females and 45 males were collected from October 2013 to December 2015 in the Bahía de La Paz, Mexico. Females ranged in size from 48.5 cm to 84 cm TL, males ranged from 41.5 cm to 58.5 cm TL. Thus, the females were larger than males in the biological samples during the study period. The sex ratio of *Narcine entemedor* showed that there was a dominance of males in the range of 45–55 cm TL; conversely, the females were more abundant from 60 cm to 85 cm TL (Figure 1). The monthly sex ratio showed a dominance of females and an absence of males was observed during January, April, and June. However, during July–September, the presence of males increased (Figure 2). Nonetheless, the sex ratio assessed from the X^2^ test (*p* < 0.05) showed that only during three months the sex ratio was 1:1. These months were March (X^2^ = 1.80, df = 1), September (X^2^ = 0.75, df = 1), and November (X^2^ = 1.80, df = 1) (Table 2).

**Figure 3:**
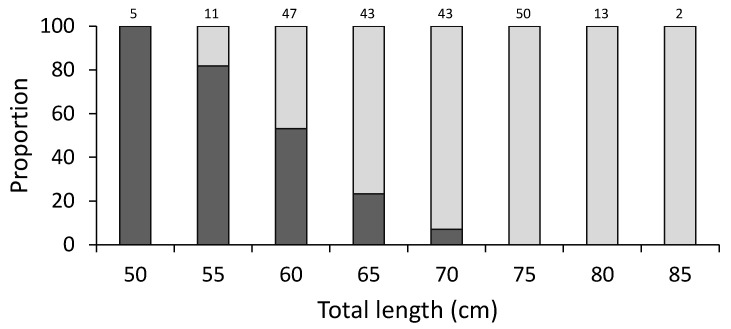
Proportion at length of immature (dark gray bars) and mature (light gray bars) females of *Narcine entemedor* throughout the study period (*n* = 214). The sample size is denoted above each bar.

**Figure 4:**
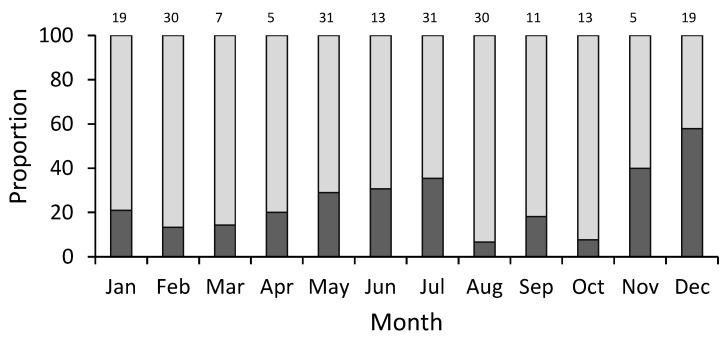
Monthly proportions of immature (dark gray bars) and mature (light gray bars) females of *Narcine entemedor* throughout the study period (*n* = 214). The sample size is denoted above each bar.

All males analyzed in the present study were mature. Of the total number of females analyzed, 17.7% were immature. The proportion of maturity, expressed as the relationship between immature and mature females, showed that the larger females of 55 cm TL were mature, and the dominance of mature females was observed from 65 cm TL. An overlap between immature and mature females was identified for individuals smaller than 70 cm TL (Figure 3). The monthly proportions of maturity showed that mature females were present all year round, with the first change in proportions of immature females observed from January to April, with high values during January–February, and low proportions during March–April. A second change in the proportion of immature females occurred with a decrease from May to September, and the third change was an increase in proportions of immature females observed from October to December (Figure 4). These results suggested that there was no seasonality in the reproductive pattern for *N. entemedor*, given than the females were mature from 55 cm TL and in high proportions throughout the year.

**Figure 5:**
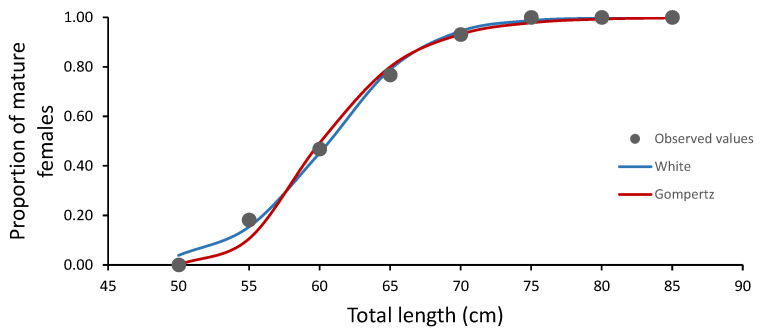
Sigmoid and logistic models (lines) fitted to the length at maturity data (points) for female giant electric rays, *Narcine entemedor*.

The estimates of length at maturity and parameters for each model are shown in Table 3. The $L_{50}$ value estimated through GOM and WHI showed a difference of approximately 2 cm, where the GOM exhibited a smaller value. The parameterization of WHI indicated that the asymptotic value expressed from *ε* was 1, indicating that the females from 60.64 cm TL progressively increased their maturity proportions at length until reaching the total length of 85 cm, although the asymptote was promptly described by both models from 65 cm. The comparison between models and the model selection between them showed that the GOM model had the maximum likelihood (73.6) and smaller AIC, indicating that this model was a better fit to the maturity proportion data of *N. entemedor*. A partial overlap between trajectories estimated that mature proportions for the two models were observed. The trajectories computed for both models showed that the GOM underestimated the maturity proportions at length for smaller lengths (55 cm TL) (Figure 5). 

## 4. Discussion

### 4.1. Length at Maturity

This study reanalyzed the length at maturity data for *N. entemedor*, mainly because an interesting feature was observed in previous studies. This feature was the symmetry of the model reported and its performance for fitting observed data, which was apparently well distributed around the model. Our results showed a lack of symmetry in the observed data fitted to the $L_{50}$ model. Commonly, the observations could be expected, assuming that the cumulative distribution function was symmetric; however, the data set provided more information close to the asymptotic value, therefore the functional form of both models and observed data were important for estimating $L_{50}$. In this study, two models were statistically compared (GOM and WHI), avoiding an analysis based on mathematical expressions yielding similar estimates of $L_{50}$, such as was documented by Oviedo-Pérez et al. [12] and García-Rodríguez et al. [16]. The estimates obtained from GOM indicated that the $L_{50}$ value for this species was similar to the length reported by Burgos-Vázquez et al. [18], while the WHI provided a larger $L_{50}$ value. This comparison suggested that the data set was distributed around the model, covering all the size classes. Whether this condition was observed or not could have caused misspecifications in the models, providing bias in $L_{50}$ estimates with evident poor fit [31,32,33]. 

Estimates of length at maturity were different between the two models used. The values associated with the Akaike information criterion indicated values of 151.16 (GOM) and 151.94 (WHI); consequently, the GOM was the best model selected using the maximum likelihood values estimated [30]. In this study, the WHI was implemented for the final estimation of $L_{50}$, the main assumption was that the reproductive event in *Narcine entemedor* was a nonlinear process related to its total length, assuming that not all mature females had reproductive activity at the same time; thus, the maximum proportion of maturity reached will be different to an asymptotic value of 1 [6] (pp. 81–127, [17]). However, this assumption was not satisfied for this species. Conversely, the proportion of maturity observed in *N. entemedor* was sufficiently informative for an asymptote equal to 1, this was clearly influenced for females larger than the 65 cm size class (TL), indicating that it was a coincident with a continuous annual reproductive cycle and the absence of a reproductive peak for mature females.

### 4.2. Features of the Reproductive Biology Affecting $L_{50}$

This analysis was supported by biological information obtained from commercial artisanal fisheries, where *N. entemedor* was not a target species. Consequently, the data were limited, nonetheless there was the biological information necessary for analyzing the basic features of reproduction for the giant electric ray. We observed that the sex ratio of *Narcine entemedor* was dominated by males with a total length less than 55 cm, while the females with a total length above 65 cm were more abundant. Females were dominant across all months observed through the annual cycle. According to Villavicencio-Garayzar [19], the males of this species are scarce; thus, the annual sex ratio estimated during 1992 was ~11:1. This change in sex ratio could be attributed to differences in the growth pattern by sex. Smith et al. [34] reported for *Hypanus dipterura*, that the age structure of the population was sexually dimorphic, in that males had a longevity of 19 years while that of the females was 28 years. A similar age structure was found for *Himatura astra*, in which the males had 19 age classes and the females had 30 age classes [35]. A similar pattern was described for *Platyrhina sinensis*, with a maximum age reported for males of 5 years and for females the respective age was 12 years [36].

The length structure of *Narcine entemedor* was also different by sex, with females being larger than males. Similarly, for *Hypanus sayi* (*H. sayi*), *Torpedo torpedo* (*T. torpedo*), and *Torpedo marmorata* (*T. marmorata*) the females attained a larger size than the males [37,38]. For *H. sayi*, the largest female had a 72.9 cm disc width (DW) and the largest male had a 52.1 cm DW [37], while for *T. torpedo*, the total length reported for females was 47.7 cm TL and for males it was 44.5 cm TL. For *T. marmorata*, the difference was also 20 cm TL (55.3 cm TL for females and 36.4 cm TL for males). Additionally, in *T. torpedo*, the sex ratio did not differ among size groups, but in T. marmorata, the presence of females exceeded males at sizes >34.1 cm TL [38]. According to Rolim et al. [39], the length structure of *Narcine brasiliensis* is different between males (from 23.6 cm to 38.0 cm LT) and females (from 23.7 cm to 47.0 cm LT). The dominance of females measured from sex ratio for the populations of rays previously referred to were always higher than males, with females attaining larger sizes.

According to Koob and Callard [40], the reproductive cycles in elasmobranchs can be classified into three types, made up of distinct species assemblages: (1) continuous breeders, (2) seasonal breeders, and (3) punctuated breeders. *Narcine entemedor* is classified as an organism with lecithotrophic viviparity. Lecithotrophy is a developmental pattern in which yolk, produced by the maternal liver and sequestered in the yolk sac, provides embryonic nutrition [17]. However, Burgos-Vázquez et al. [18] suggested that the giant electric ray presented limited histotrophy as a reproductive mode and has a continuous annual reproductive cycle; one peak of ovulation occurs between July and September, but two peaks of parturition occur (minor peak in January–February and major peak in August–September). In the Bahía de La Paz, the reproductive period of *Narcine entemedor* was not temporally defined because the presence of a reproductive peak for mature females was not observed from the mature/immature ratio. This feature could be associated to its northernmost distribution zone, where the environmental conditions (e.g., food, temperature) have an influence on its population structure and reproductive strategy. 

Species with lecithotrophic oviparity, in which a continuous annual reproductive cycle has been reported, are relatively commonly. These include *Raja clavata* (Gulf of Gabés) with an absence of a reproductive peak [41]. This species attains maturity at a younger age off the Strait of Sicily, meanwhile in North Wales the maturity is commonly observed at an older age [42,43]. Conversely, *Leucoraja naevus* (*L. naevus*) has a reproductive peak in Southern European waters (January–May), Celtic Seas (February), and the North Sea (September–December) [44]. Additionally, both species exhibit a latitudinal gradient in size structure: *Raja clavate* (*R. clavate*) was larger in the Celtic Seas (98 cm TL) than in the North Sea (92 cm TL). For *L. naevus*, the maximum total length was 69 cm in the Celtic Seas and 62 cm TL in the North Sea [45].

The length at maturity of *N. entemedor* off the southwest coast of the Baja California Peninsula (Bahía Magdalena) was reported to vary between 62 cm and 63 cm TL. This length interval represented approximately 68% of its maximum total length [19]. Meanwhile, Burgos-Vázquez et al. [18] estimated a value of 58.5 cm TL (CI = 51.7–65.4 cm TL) in the Bahía de la Paz (Gulf of California) using a logistic regression model. In this study, the length at maturity was less than that previously reported by Villavicencio-Garayzar [19]. This value was supported by a sigmoid model (GOM) with a different trajectory in comparison to WHI. The advantage of the GOM was that it had a more flexible form; it had a rapid inflexion point in the first length classes, showing a slower approach to the maximum fraction of mature females (asymptotic value). Thus, the comparison among the length-at-maturity models more frequently used and reported in the literature showed that the GOM fitted to the data was better than the logistic model.

For several batoid populations, changes in length structure and $L_{50}$ estimates have been found, mainly through latitudinal gradients. For *N. entemedor*, differences were found in the Equatorial zone, where this species attains larger sizes (110 cm TL) and $L_{50}$ = 70 cm TL [46]. This study reported lower values, with the maximum length being 84 cm TL and $L_{50}$ = 55.8 cm TL. Moreover, *Raja clavata* distributed in the Atlantic Ocean from Iceland to southern Africa [47] has differences in $L_{50}$ estimates. According to McCully et al. [45], the females inhabiting the North Sea showed the values $L_{50}$ = 73.7 cm and 77.1 cm TL, versus those from the Black Sea varying between $L_{50}$ = 66.7 cm and 74.6 cm TL. Similar results were reported for *Leucoraja naevus* distributed from Norway to Morocco and Tunisia, including in the Mediterranean Sea [48]. McCully et al. [45] found significant statistical differences between estimates from the North Sea ($L_{50}$ = 53.6 cm TL) and the Celtic Sea ($L_{50}$ = 59.8 cm TL).

## 5. Conclusions

In conclusion, considering that *Narcine entemedor* is distributed from the northwest Mexican Pacific to Peru, the population in this study inhabited the northernmost limit for the species. Therefore, reproductive biology values were different from populations elsewhere. This included sex ratio, proportions of maturity, and length at maturity over a year’s period. This species did not appear to have a reproductive peak and had a continuous annual reproductive cycle. The estimates of $L_{50}$ for this species showed that a sigmoid model (GOM) was better than the logistic model.

## Figures and Tables

**Table 1:** Candidate length-at-maturity models used to estimate $L_{50}$ for *Narcine entemedor*.

Model	Abbreviation	Function	Source
Gompertz	GOM	$P_{i}=exp^{-exp^{-\gamma (TL_{i}-L_{50}})}$	[8]
White	WHI	$P_{i}=\frac{\varepsilon }{1+exp^{\left[-\ln\left(19\right)\frac{\left(TL_{i}-L_{50}\right)}{\left(L_{95}-L_{50}\right)}\right]}}$	[10]

**Table 2:** Monthly values of chi-squared (X^2^) test estimated for *Narcine entemedor* from the Bahía de La Paz, Baja California Sur, Mexico.

	Female	Male	X^2^
January	21	0	10.50
February	37	4	13.28
March	8	2	1.80
April	8	0	4.00
May	35	3	13.47
June	17	0	8.50
July	33	13	4.35
August	33	10	6.15
September	15	9	0.75
October	16	1	6.62
November	8	2	1.80
December	29	1	13.07

**Table 3:** Parameters (in bold) and confidence intervals (in parenthesis) estimated from negative ln-likelihood contours (*p* < 0.05). $L_{50}$ is the length at which 50% of the organisms were mature, $L_{95}$ is the length at which 95% of the organisms are mature.

Model	*L_50_*	γ	*L_95_*	ε	−lnℒ	AIC
GOM	**58.50** (56.2–60)	**0.231** (0.16–0.31)			73.585	151.16
WHI	**60.64** (58.6–62.2)		**70.37** (67.6–74.4)	1	72.970	151.94

## Data Availability

Not applicable.
